# Mo^6+^ activated multimetal oxygen-evolving catalysts†
†Electronic supplementary information (ESI) available: SEM and TEM images, XANES spectra and extra electrochemical test data. See DOI: 10.1039/c6sc04819f



**DOI:** 10.1039/c6sc04819f

**Published:** 2017-02-17

**Authors:** Peng Fei Liu, Shuang Yang, Li Rong Zheng, Bo Zhang, Hua Gui Yang

**Affiliations:** a Key Laboratory for Ultrafine Materials of Ministry of Education , School of Materials Science and Engineering , East China University of Science and Technology , Shanghai , 200237 , China . Email: hgyang@ecust.edu.cn; b Department of Physics , East China University of Science and Technology , Shanghai , 200237 , China; c Department of Electrical and Computer Engineering , University of Toronto , 35 St George Street , Toronto , Ontario M5S 1A4 , Canada; d Beijing Synchrotron Radiation Facility , Institute of High Energy Physics , Chinese Academy of Sciences , Beijing , 100049 , China

## Abstract

Water splitting is key to electrically-powered chemical fuel synthesis, but the slow kinetics of the oxygen evolution reaction (OER) hinder the wider promotion of such technology. Several first-row (3d) transition metal-based catalysts have been developed for the OER; however, these catalysts still require operating voltages that lie well above the fundamental thermodynamic potential. Here, we report high-valence metal molybdenum (Mo^6+^) modulated 3d metal (oxy)hydroxides. The obtained multimetal FeCoMo based OER catalysts require an overpotential of 277 mV to reach the current density of 10 mA cm^–2^ on the glassy carbon electrode, and there was no evidence of degradation for about 40 hours of stability testing. The catalysts stay in their amorphous phases, potentially with atomically homogenous metal distribution. The *in situ* X-ray adsorption analysis unambiguously reveals the tuned electronic structures of the 3d metals owing to Mo^6+^, further demonstrating the modification effect of a high-valence metal for designing highly-efficient OER catalysts.

## Abstract 2

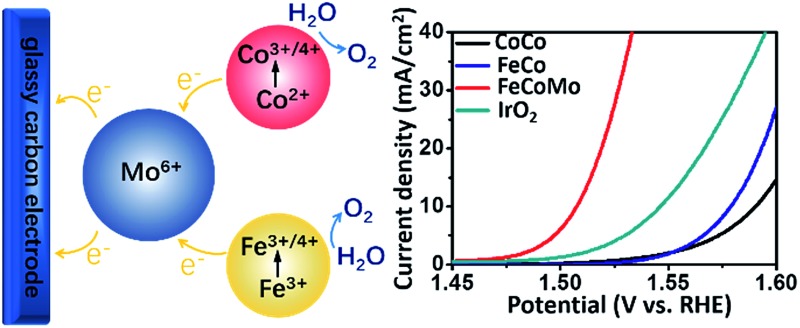
An amorphous multimetal FeCoMo OER catalyst has been successfully synthesized, activated by high-valence Mo^6+^, evidencing superior performance over benchmarking IrO_2_.

## Introduction

Oxygen electrochemistry plays a vital role in energy-related technologies, such as fuel cells, and water splitting and CO_2_ reduction electrolyzers.^1–5^ Unfortunately, the commercialization of these devices is strongly hampered by challenges in developing efficient and durable catalysts to motivate the sluggish oxygen evolution reaction (OER).^3,6^ Recently, 3d transition metal based oxides, including (oxy)hydroxides,^7–10^ perovskite oxides,^11,12^ cobalt phosphates^3,13,14^ and nickel borates,^15^ have been identified as high-activity OER catalysts. Known approaches like nanostructuring and alloying have been widely used to increase the surface area or improve the intrinsic activity of such materials, with nanostructured NiFe based oxides being the most explored and active catalysts.^16–19^ Nevertheless, new design strategies are still urgently needed to explore high-performance and cost-effective OER catalysts.

Alloyed amorphous oxides have been suggested as active catalysts for the OER, owing to their unique structures with abundant defect active sites.^20–22^ Furthermore, the high-valence metal tungsten (W) has been subtly incorporated into alloyed oxides to form homogeneously dispersed multimetal catalysts.^23^ The electronic structures of 3d metals were systematically modulated by W^6+^ to reach an optimized adsorption energy for the OER intermediates. Following this design strategy, we anticipate other high-valence elements would also activate classical 3d metal alloyed oxide OER catalysts. Thus, we examined molybdenum (Mo) tuned multimetal OER catalysts, in which Mo is a versatile coordination host in its highest valence state.

Herein, we have synthesized amorphous Mo^6+^ hosted FeCo (oxy)hydroxides (FeCoMo), exhibiting extraordinary OER performance compared to those of binary FeCo (oxy)hydroxides (FeCo) and unary Co (oxy)hydroxides (CoCo). The FeCoMo catalyst produces a current density (*j*) of 10 mA cm^–2^ at an overpotential (*η*) of 277 mV, with a large bulk mass activity of 177.35 A g^–1^ at an *η* value of 300 mV, which is nearly 7 times higher than that of the benchmarking IrO_2_ electrocatalyst. At the same time, the ternary OER catalyst could maintain a current density of 10 mA cm^–2^ for about 40 hours (h) with no obvious degradation. In general, the high-valence Mo^6+^ activates the classical (oxy)hydroxide OER catalysts, with rich active sites for a successful OER process. We believe that high-valence element induced amorphous alloyed OER catalysts would provide prospective insights into designing highly effective water oxidation electrocatalysts.

## Results and discussion

**Fig. 1:**
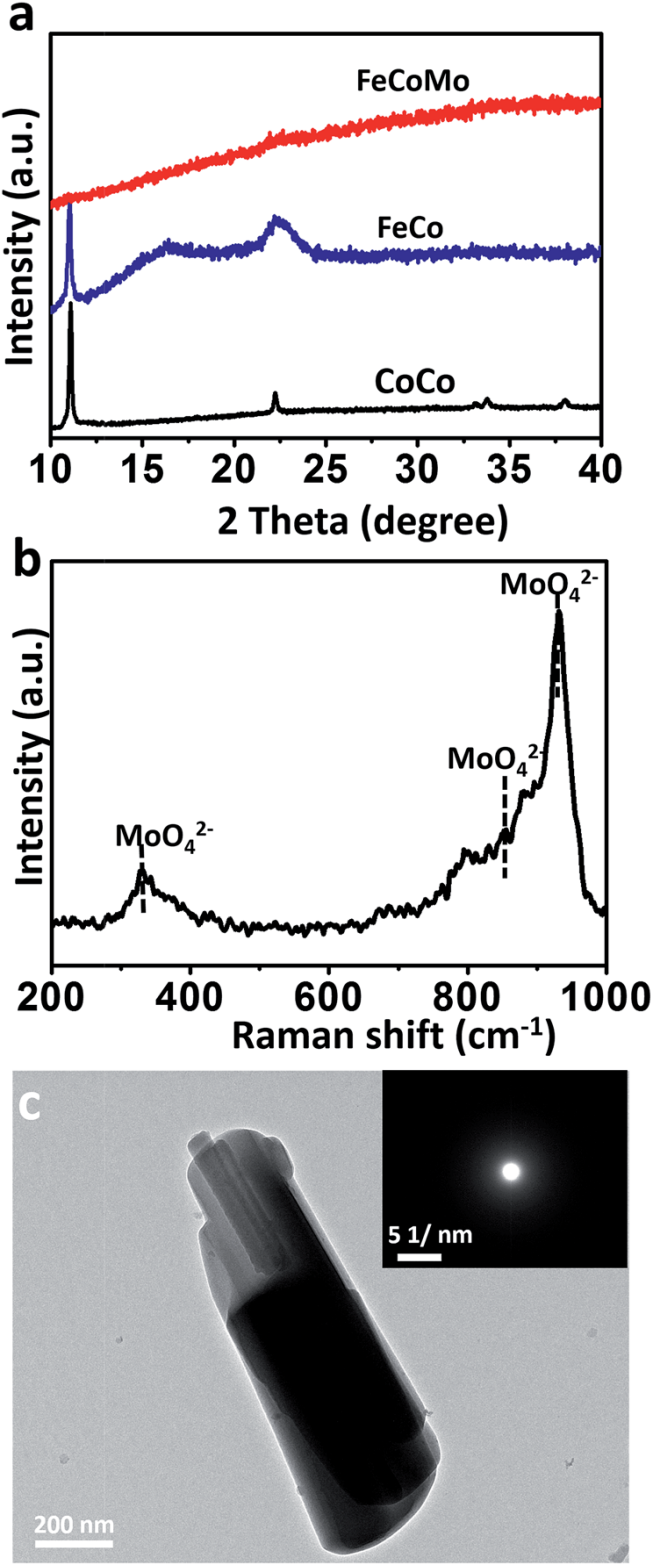
(a) The XRD patterns for the samples of unary CoCo, binary FeCo and ternary FeCoMo (oxy)hydroxides. (b) The Raman spectrum of the FeCoMo sample. (c) The TEM image of the FeCoMo sample. The inset of (c): the SAED pattern of the FeCoMo sample.

To synthesize the FeCoMo samples, cobalt chloride hexahydrate (CoCl_2_·6H_2_O) and iron chloride hexahydrate (FeCl_3_·6H_2_O) were continuously hydrolysed with the addition of ammonium molybdate tetrahydrate ((NH_4_)_6_Mo_7_O_24_·4H_2_O) and hexamethylene tetramine (HMT) at a temperature of 90 °C. Because pyrolyzed (NH_4_)_6_Mo_7_O_24_ and HMT slowly generate MoO_4_^2–^ and OH^–^ simultaneously,^24,25^ small fragments like metal molybdates and hydroxides were irregularly precipitated,^26^ and then the amorphous phases with long range ordering were obtained. As shown in Fig. 1a, X-ray diffraction (XRD) patterns revealed that the ternary FeCoMo samples stayed in the amorphous phase, without any obvious diffraction peaks. For comparison, the binary FeCo and unary CoCo samples had a layered double hydroxide (LDH) composition. The digital images of these samples are shown in Fig. S1,† and all three samples exhibited different colors. The Raman spectrum (Fig. 1b) further demonstrated the existence of thermolysis derived MoO_4_^2–^ ions, with featured bands at 330 cm^–1^ (bending modes), 855 cm^–1^ (antisymmetric stretching mode) and 932 cm^–1^ (symmetric stretching modes).^27^

To characterize the morphologies of the FeCoMo sample and the controlled samples, scanning electron microscopy (SEM) and transmission electron microscopy (TEM) were carried out. In Fig. S2,† the CoCo sample displayed a plate-like shape, while the other two samples exhibited no particular morphology. In order to identify the uniformity of the FeCoMo catalyst, energy dispersive spectroscopy (EDS) element mapping was conducted. In Fig. S3,† the elements Fe, Co and Mo were uniformly distributed on the FeCoMo sample. In addition, the low-magnification TEM images of the FeCoMo sample showed rod-like shapes (Fig. 1c and S4†). Furthermore, the selected area electron diffraction (SAED) analysis again confirmed the amorphous structure of the FeCoMo sample (the inset of Fig. 1c), without any crystalline phases, which was consistent with the XRD results.

**Fig. 2:**
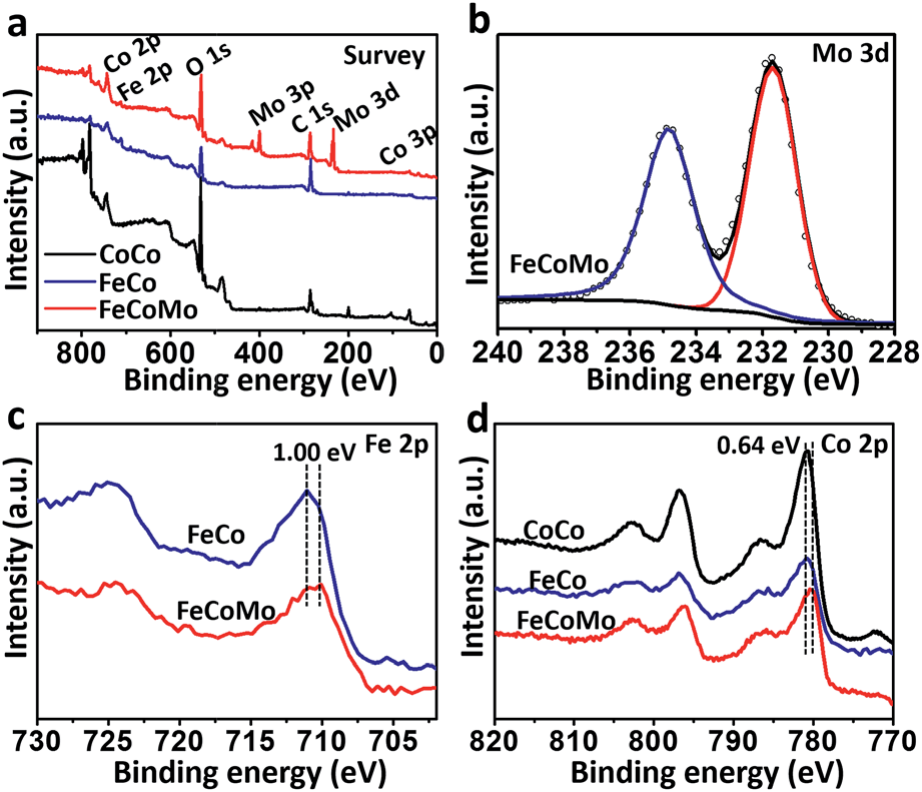
(a) The survey XPS spectra for the samples of unary CoCo, binary FeCo and ternary FeCoMo. (b) The XPS spectrum of the Mo 3d region for the FeCoMo sample. (c) The XPS spectra of the Fe 2p region for the samples of FeCo and FeCoMo. (d) The XPS spectra of the Co 2p region for the samples of CoCo, FeCo and FeCoMo.

In order to compare the differences in the surface electronic structure for these three samples, X-ray photoelectron spectroscopy (XPS) was then carried out. In Fig. 2a, the survey XPS spectra proved that the Co element existed in all three samples, with additional Fe in FeCo and FeCoMo, and Mo in the FeCoMo sample. In the XPS spectrum of the Mo 3d region for FeCoMo (Fig. 2b), two apparent peaks at 231.7 and 234.8 eV can be observed, which were assigned to the Mo^6+^ species, indicating that the Mo element was in its highest valence state on the surface of the FeCoMo sample.^28^ In the XPS spectra of the Fe 2p region (Fig. 2c), the featured peaks of FeCoMo were shifted to a lower binding energy relative to the FeCo sample (∼1.0 eV), which suggested that the Fe element was in a relatively lower valence state in the FeCoMo sample, further demonstrating the strong electronic interactions between Fe and Mo. Meanwhile for the Co 2p region, the surface valence states of CoCo and FeCo behaved similarly to one another (Fig. 2d). For comparison, cobalt’s valence state in FeCoMo was slightly lower than in the other two samples.

To detect the bulk electronic structures, X-ray absorption near edge structure (XANES) spectra were collected, and the corresponding pre-edges are shown in Fig. S5.† The data showed that the three samples mainly consisted of Fe^3+^ in the bulk. A similar distribution was found for Co, and cobalt’s valence state exhibited negligible difference for the three samples. In particular, the bulk valence states of Co in the samples were lower than that of Co_3_O_4_.

**Fig. 3:**
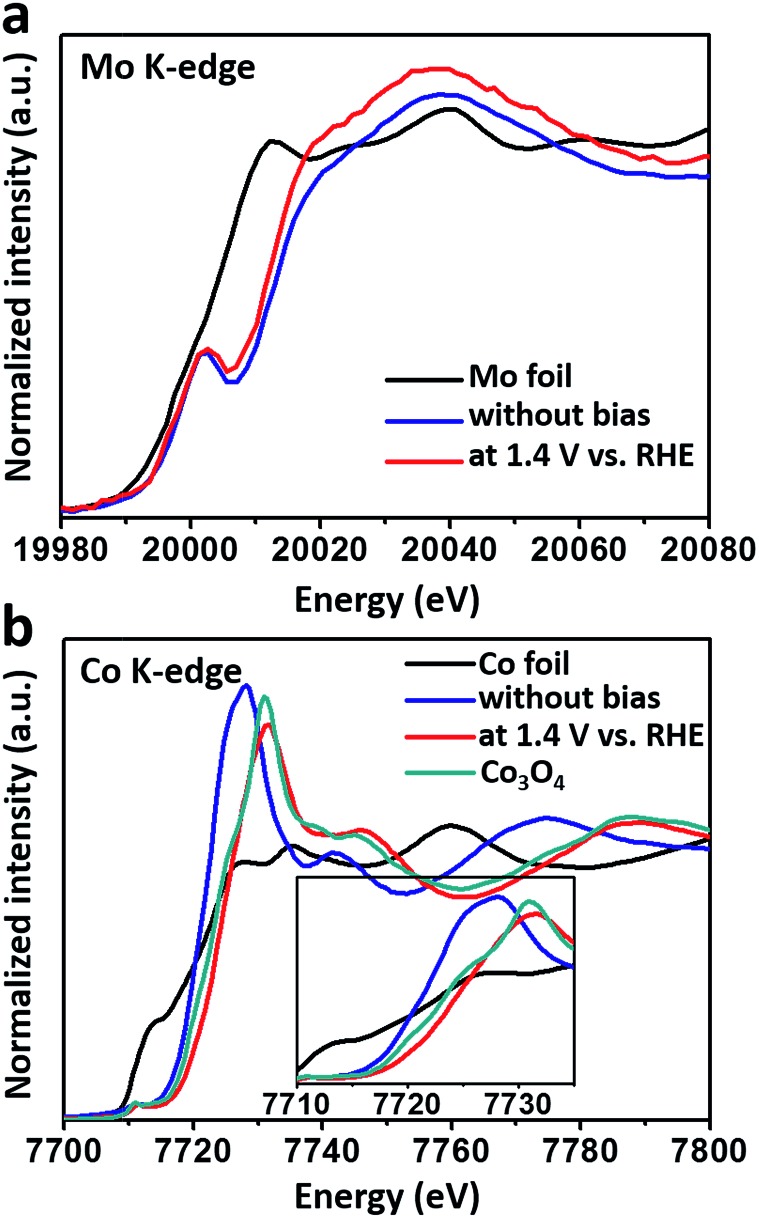
The *in situ* XANES spectra of (a) the Mo K-edge and (b) the Co K-edge for the FeCoMo sample with and without an applied bias (1.4 V *vs.* RHE). The inset of (b): the zoomed in pre-edges of the Co K-edge.

To monitor the bulk valence changes of the introduced versatile Mo^6+^ and 3d transition metals, *in situ* XANES tests were carried out, simultaneously testifying the modulation effect of high-valence metals in the real OER environment. In Fig. 3a, the XANES results of the Mo K-edge showed a continuous decrease in the energy of the absorption edge (∼1.4 eV) when a potential of 1.4 V (*vs.* RHE) was applied, indicating that the valence of Mo was decreased under the OER process. Meanwhile, the increased intensity of the white line suggested the decrease in the distorted octahedral environment around the Mo atom.^29^ In contrast, the valence of Co inversely increased under the water oxidation conditions (Fig. 3b), and was higher than that of Co_3_O_4_, nearly resembling that of active CoOOH.^7^ While for the Fe K-edge, iron’s valence in the FeCoMo sample nearly stayed the same as Fe^3+^ under the oxidation conditions (Fig. S6†). Notably, the decreased intensity of the white line for both Co and Fe evidenced the more distorted octahedral structure upon oxidation.^30,31^ Recent studies have proposed that high-valence Fe species, with unusually shorter Fe–O bonds and distorted octahedral structures, could probably be regarded as active sites for the OER, as evidenced using *in situ* analysis technologies.^32–35^ Although the high-valence Fe (Fe^4+^) has not been characterized in our experiments, we still think that the distorted FeO_6_ octahedra might contribute to the OER process. Overall, the Mo^6+^ species play an effective role in tuning the electronic structures of 3d metals. When applied under oxidation conditions, the external potential would stimulate the electrons of the 3d metals to transfer to Mo^6+^, which was in the highest oxidation state. Benefiting from the prominent ability of high-valence Mo^6+^ to draw electrons, the 3d metals would easily stay in their high-valence states, which are widely regarded as active sites for the OER.

**Fig. 4:**
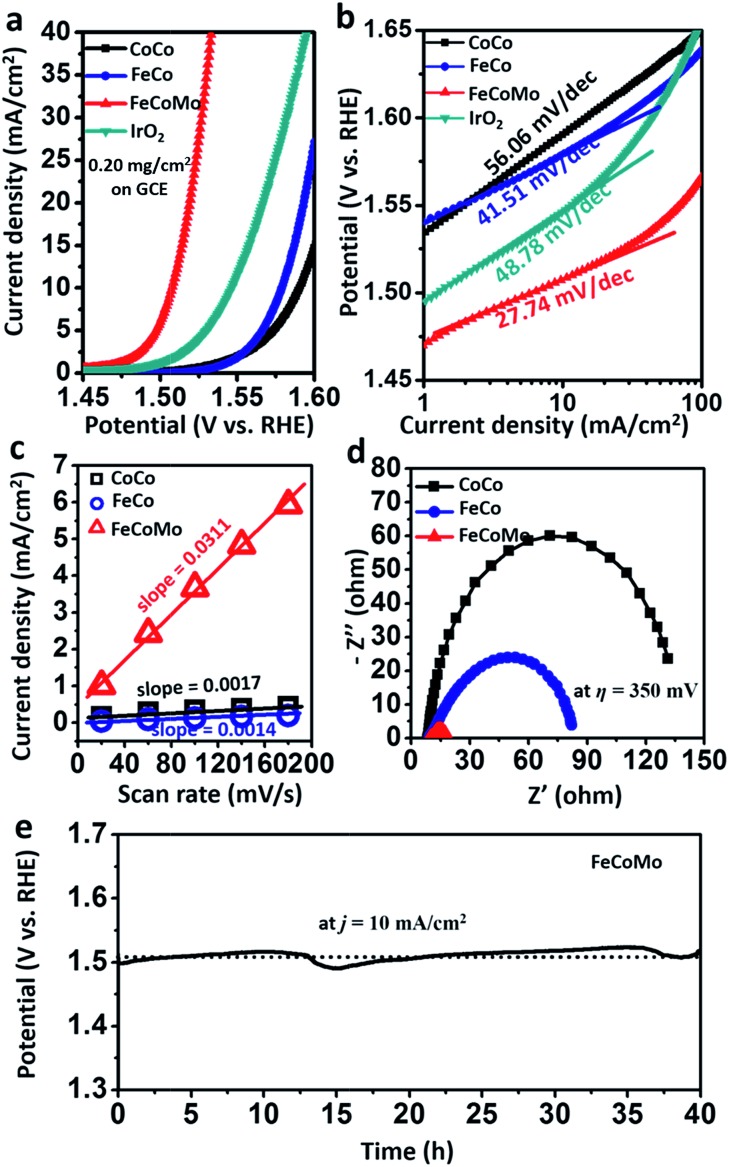
(a) The LSV curves and (b) the corresponding Tafel slopes of the CoCo, FeCo, FeCoMo and benchmarking IrO_2_ samples. (c) Scan rate dependence of the current densities for CoCo, FeCo and FeCoMo at 0.25 V *vs.* Ag/AgCl/3.5 M KCl. (d) Nyquist plots of CoCo, FeCo and FeCoMo at an *η* of 350 mV. (e) Chronopotentiometric curves of FeCoMo at a *j* of 10 mA cm^–2^ for the continuous OER process.

To evaluate the OER activities of amorphous multimetal FeCoMo catalysts, all of the electrocatalytic tests were conducted *via* a three-electrode system in an H-type cell with 1 M KOH electrolyte. In Fig. 4a and S7,† the FeCoMo catalyst required an *η* of 277 mV, 289 mV, and 336 mV to achieve a *j* of 10 mA cm^–2^, 20 mA cm^–2^ and 100 mA cm^–2^, respectively, which outperformed CoCo, FeCo and commercial IrO_2_ catalysts. The OER performances of the annealed samples were also evaluated, as shown in Fig. S8.† The amorphous FeCoMo catalyst exhibited better activity than the annealed one. To analyze the electrokinetics of the catalysts, Tafel plots were directly derived from the linear sweep voltammetry curves that were fit to the Tafel equation (*η* = *b* log *j* + *a*, where *b* is the Tafel slope). As shown in Fig. 4b, FeCoMo has a Tafel slope of 27.74 mV dec^–1^, which is much lower than those of CoCo (56.06 mV dec^–1^), FeCo (41.51 mV dec^–1^) and IrO_2_ (48.78 mV dec^–1^). Considering that the Tafel slope has been traditionally used to infer which of the four electron-/proton-transfer steps are rate limiting in the OER process, the Tafel slope of FeCoMo which is near 24 mV dec^–1^ indicates that the third electron transfer step is rate limiting.^29^ Furthermore, FeCoMo’s Tafel slope is very close to those observed for excellent Ni(Fe)OOH thin films (25–40 mV dec^–1^) in previous reports, implying that a similarly efficient OER mechanism exists. In general, the amorphous FeCoMo catalysts demonstrate excellent OER performances compared to benchmarking IrO_2_ and other controlled (oxy)hydroxides reported in the literature (Tables 1 and S1†).

**Table 1:** Comparison of the OER catalytic parameters of FeCoMo and controlled (oxy)hydroxides

Catalysts^*a*^	*η*_10_ ^*b*^ (mV)	*j*_300_ ^*c*^ (mA cm^–2^)	Tafel slope (mV dec^–1^)	Bulk mass activity^*d*^ (A g^–1^)	References
FeCoMo	277	35.5	27.74	177.35	This work
CoCo	361	0.9	56.06	4.40	This work
FeCo	349	0.5	41.51	2.30	This work
IrO_2_	316	5.0	48.78	25.20	This work
CoOOH	300	10	38	66.6	7
α Ni(OH)_2_	331	∼5	∼42	∼10	8
NiFe nanosheets	302	∼9.4	40	∼134.29	9
NiV nanosheets	318	∼5	∼50	∼34.97	10
G-FeCoW	223	246.8	37	1175	23

^*a*^All of the catalysts were deposited on the GCE to evaluate their OER performances.

^*b*^ *η*_10_ is the overpotential to achieve a current density of 10 mA cm^–2^.

^*c*^ *j*_300_ is the current density at an overpotential of 300 mV.

^*d*^The bulk mass activity was calculated at an overpotential of 300 mV.

In order to compare the electrochemical surface areas (ECSAs) of the FeCoMo and controlled samples, the double-layer capacitances (*C*_dl_) of the samples were estimated using a simple cyclic voltammetry (CV) method. As shown in Fig. S9† and 4c, the plots of Δ*j* = (*j*_a_ – *j*_c_) at 0.25 V (*vs.* Ag/AgCl/3.5 M KCl) against the scan rates were recorded. We can clearly find that the slope (equivalent to twice the value of *C*_dl_) of FeCoMo (0.0311 F cm^–2^) is much larger than that of CoCo (0.0017 F cm^–2^) and that of FeCo (0.0014 F cm^–2^), suggesting that the FeCoMo catalyst had more exposed active sites for the OER. Next, we further investigated the intrinsic activity when the OER activity was normalized using Brunauer–Emmett–Teller (BET) surface areas, which is appropriate for these traditional (oxy)hydroxide catalysts with poor conductivity when they are not completely oxidized.^36,37^ In Fig. S10,† the intrinsic activity of the amorphous FeCoMo sample was notably higher than the controlled sample, with a large *j* (normalized using the BET surface areas) of 1.4 mA cm^–2^ at an *η* of 300 mV. It is worth mentioning that the BET surface area of the FeCoMo catalyst is only 10.5 m^2^ g^–1^, and its OER performance can be improved when the material is more nanostructured.

Electrical impedance spectroscopy (EIS) was carried out to evaluate the electric properties of the FeCoMo and controlled samples. In Fig. 4d, the FeCoMo catalyst showed the lowest transport resistance, indicating that it had superior charge transport kinetics.

To estimate the stability of the FeCoMo catalyst, we ran a chronopotentiometric test on the water oxidation of the catalyst with a constant *j* of 10 mA cm^–2^. In Fig. 4e, the potential of FeCoMo maintained around 1.5 V for about 40 h, without any appreciable deactivation, further demonstrating the operating stability of FeCoMo for large application. For comparison, the IrO_2_ benchmark catalyst exhibited poor stability (Fig. S11†). Moreover, we collected the FeCoMo sample after the stability tests and characterized it using SAED analysis, and the FeCoMo catalyst remained in the amorphous phase (Fig. S12†). To confirm whether the high-valence Mo^6+^ would be leached, inductively coupled plasma atomic emission spectroscopy (ICP-AES) measurements were conducted and almost no leaching phenomenon was observed (Table S2†). Furthermore, the EDS mapping images of FeCoMo after the stability tests also indicated that negligible Mo^6+^ species was leached (Fig. S13†).

## Conclusions

In summary, Mo^6+^ activated amorphous multimetal oxygen-evolving catalysts have been successfully synthesized. The electronic structures of 3d transition metals in metal (oxy)hydroxides have been subtly modulated by high-valence metal Mo^6+^, to reach an optimized adsorption energy for the OER intermediates. The *in situ* XANES data have unambiguously identified that Mo^6+^ tends to draw in electrons to increase the valence of the 3d metals for promoting the OER. We believe that a high-valence metal incorporation strategy would pave a new way for rationally designing effective multimetal OER catalysts, holding promise for cheap, efficient and large scale alkaline water splitting in industry.
