# Supplementary material for: Mo6+ activated multimetal oxygen-evolving catalysts
Source: Chem Sci. 2017 Feb 17;8(5):3484–8. doi: 10.1039/c6sc04819f (PMC5418644; doi:10.1039/c6sc04819f)
Supplement: Supplementary file 1 [file SC-008-C6SC04819F-s001.pdf]

Electronic Supplementary Material (ESI) for Chemical Science.
This journal is © The Royal Society of Chemistry 2017

Electronic Supplementary Information

Mo^{6+} activated multimetal oxygen-evolving catalyst

Peng Fei Liu,[a] Shuang Yang,[b] Li Rong Zheng,[d] Bo Zhang[b,c] and Hua Gui Yang[a*]

[a] Key Laboratory for Ultrafine Materials of Ministry of Education, School of Materials Science and Engineering, East China University of Science and Technology, Shanghai, 200237 (China)

[b] Department of Physics, East China University of Science and Technology, Shanghai, 200237 (China)

[c] Department of Electrical and Computer Engineering, University of Toronto, 35 St George Street, Toronto, Ontario M5S 1A4 (Canada)

[d] Beijing Synchrotron Radiation Facility, Institute of High Energy Physics, Chinese Academy of Sciences, Beijing, 100049 (China)

[*] Corresponding author: hgyang@ecust.edu.cn (H. G. Y).

Experimental Section

Chemicals. Cobalt (II) chloride hexahydrate ($CoCl_2·6H_2O$), iron (III) chloride hexahydrate ($FeCl_3·6H_2O$), ammonium molybdate tetrahydrate ($(NH_4)_6Mo_7O_{24}·4H_2O$) and hexamethylene tetramine (HMT) were obtained from Sinopharm Chemical Reagent Co. Ltd. Nafion (5 wt%) was obtained from Sigma-Aldrich. Iridium (IV) oxide (IrO_2) was obtained from Strem Chemicals, Inc. All chemicals were of analytical grade and used without any further purification.

Synthesis of amorphous ternary FeCoMo sample. $CoCl_2·6H_2O$ (1.190 g), $FeCl_3·6H_2O$ (0.676 g), $(NH_4)_6Mo_7O_{24}·4H_2O$ (0.883 g) and HMT (2.00 g) were successively dissolved into a flask, which consists of 100 mL mixed solution of ultrapure water (90 mL) and anhydrous ethanol (10 mL). After that, the mixed solution was placed at the temperature of 90 ^oC under continuous stirring for 10 h. Resulting products were centrifuged at 6000 rpm for collection, and then washed with ultrapure water and anhydrous ethanol for several times.

Synthesis of unary CoCo sample. The synthetic procedure was similar to the synthesis of FeCoMo except that the precursors were without addition of $FeCl_3·6H_2O$ and $(NH_4)_6Mo_7O_{24}·4H_2O$.

Synthesis of binary FeCo sample. The synthetic procedure was similar to the synthesis of FeCoMo except that the precursors were without addition of $(NH_4)_6Mo_7O_{24}·4H_2O$.

Synthesis of annealed samples. To produce the annealed samples, the obtained CoCo, FeCo and FeCoMo samples were annealed at 500 °C for 2 h in air.

Electrochemical measurements. All the electrochemical tests for OER were performed in a conventional three-electrode system at an electrochemical station (CHI 660E), using Ag/AgCl (3.5 M KCl solution) electrode as the reference electrode, a graphite rod (spectral purity, 3 mm in diameter) as the counter electrode. To prepare the catalysts deposited on the glassy carbon electrode (GCE), 5 mg of different samples and 80 μL of Nafion solution were dispersed in 1 ml dispersions consisting of 4:1 v/v water/ethanol, and then sonicated for at least 30 min to obtain a homogeneous ink solution. After that, about 3 μL of the ink solution was carefully deposited onto the a GCE (3 mm in diameter). The final loading for all samples on the

GCE is about 0.20 mg/cm^2. Before OER test, the catalysts were cycled 20 times using cyclic voltammetry (CV) to fully activate the catalyst at OER condition. Linear sweep voltammetry with scan rate of 5 mV/s was conducted in 1 M KOH (pH 13.6) purged by Ar for 30 min at room temperature. All potentials were referenced to reversible hydrogen electrode (RHE) by following calculations: $E_{RHE} = E_{Ag/AgCl} + 0.059 \times pH + 0.205$. AC impedance measurements were carried out in the same configuration when the working electrode was biased at a certain overpotential from 10^5 Hz to 0.1 Hz with an AC voltage of 5 mV. All LSV polarization curves and chronopotentiometric curves were corrected with *iR*-compensation, and the equivalent series resistance (R_s) can be obtained from the EIS Nyquist plot as the first intercept of the main arc with the real axis. The stability test was conducted at the constant j of 10 mA/cm^2 for OER.

Bulk mass activity (A/g) values were calculated from the electrocatalysts loading m (0.20 mg/cm^2) and the measured current density j (mA/cm^2) at the overpotential η of 300 mV:

$$Bulk\ mass\ activity = j\ /\ m$$

The ECSAs were determined by measuring the capacitive current associated with double-layer charging from the scan-rate dependence of cyclic voltammograms (CVs). The potential window of cyclic voltammograms was 0.20 to 0.30 V *vs.* Ag/AgCl (3.5 M KCl solution). The double layer capacitance (C_{dl}) was estimated by plotting the $\Delta j = (j_a - j_c)$ at 0.25 V against the scan rate. The liner slope is twice of the double layer capacitance C_{dl}.

XAFS experimental details. Fe, Co and Mo K-edge absorption spectra were performed on the 1W1B beamline of the Beijing Synchrotron Radiation Facility, China, operated at ~200 mA and ~2.5 GeV. The monochromator energy was calibrated with Fe, Co and Mo foil rising edge energy, respectively.

Ex-situ samples: The spectra were directly recorded in transmission mode. We ran the Fe K-edge EXAFS in the range 6895.8 eV to 7723.8 eV, Co K-edge range in the range 7512.9 eV to 8569.0 eV and Mo K-edge in the range 19802.5 eV to 20856.3 eV with a step-size of 0.50 eV at the near edge. All the samples were prepared by placing a small amount of homogenized powder on 3M tape.

In-situ samples: The spectra were recorded in fluorescence mode. Before test, all the samples were prepared by spin-coating onto the fluorine doped tin oxide (FTO) glass to form a uniform film. All the *in-situ* spectra were collected with or without a bias in

a home-made three-electrode cell with 1 M KOH as the electrolyte. The energy range and step-size energy of Fe K-edge, Co K-edge and Mo K-edge were the same as the *ex-situ* characterization.

Characterizations. The morphologies and structures of the samples were characterized by scanning electron microscopy (SEM, Hitachi S4800) and transmission electron microscopy (TEM, JEM 2100, 200 kV). The crystal structure was determined by micro-Raman spectroscopy (Renishaw, inVia Reflex) and X-ray diffraction (XRD, D/max2550V). Furthermore, the chemical states of the elements in catalysts were studied by X-ray photoelectron spectroscopy (XPS, Kratos Axis Ultra DLD), and the binding energy of C 1s peak at 284.8 eV was taken as an internal standard. The Mo content in the electrolyte was determined by inductively coupled plasma atomic emission spectroscopy (ICP-AES, Varian 710ES). Brunauer-Emmett-Teller (BET) surface area measurement was performed at 77 K on a Micromeritics ASAS 2460 adsorption analyzer in N_2-adsorption mode.

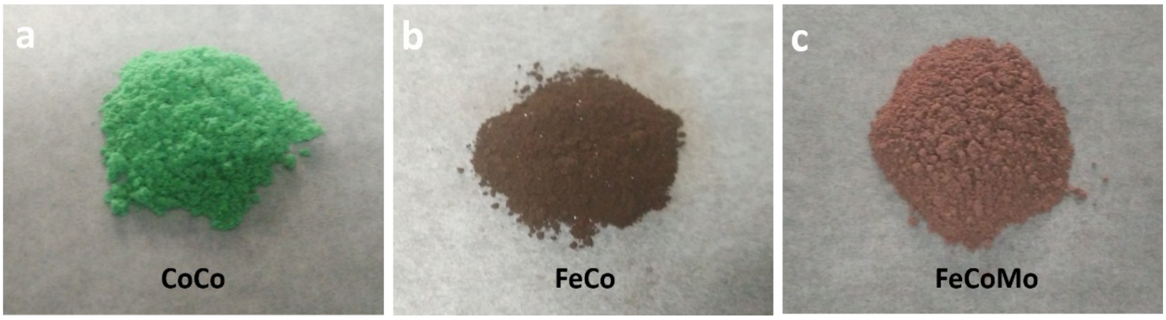

Fig. S1 Digital images of (a) CoCo sample (green), (b) FeCo sample (brown) and (c) FeCoMo sample (reddish brown).

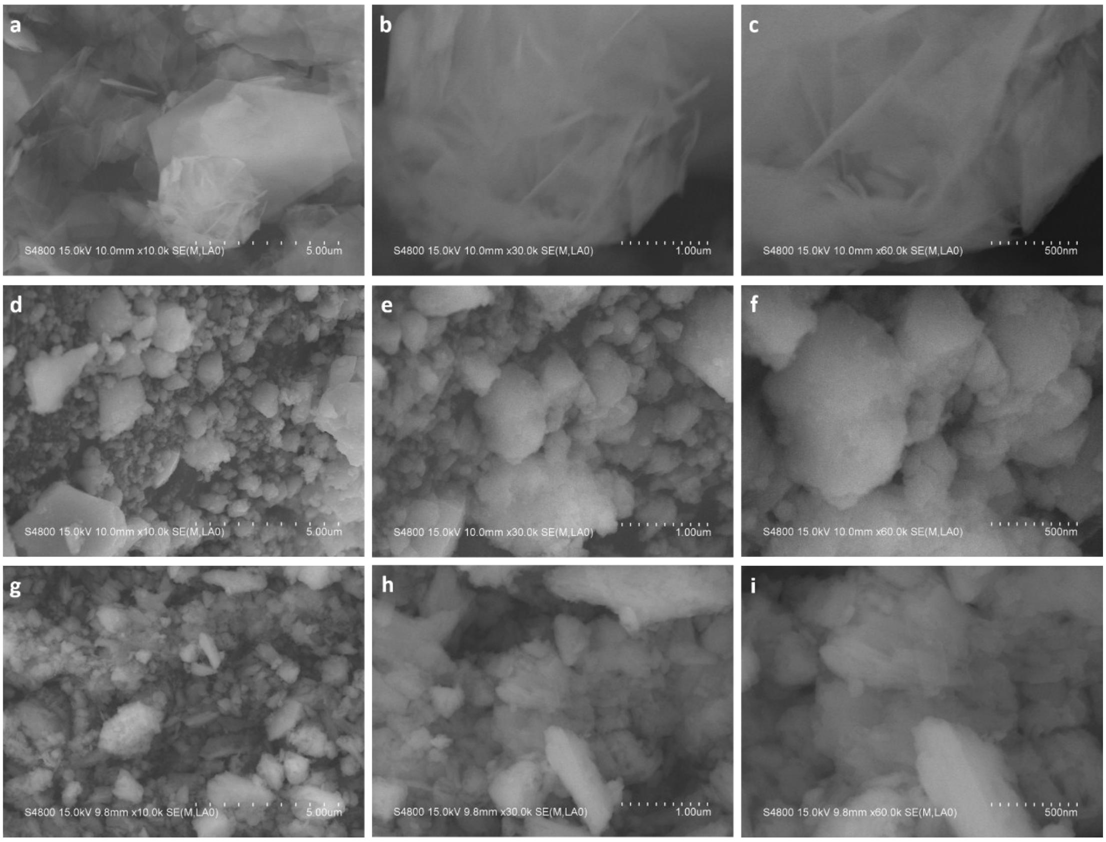

Fig. S2 SEM images of (a-c) CoCo samples, (d-f) FeCo samples and (g-i) FeCoMo samples at different magnifications.

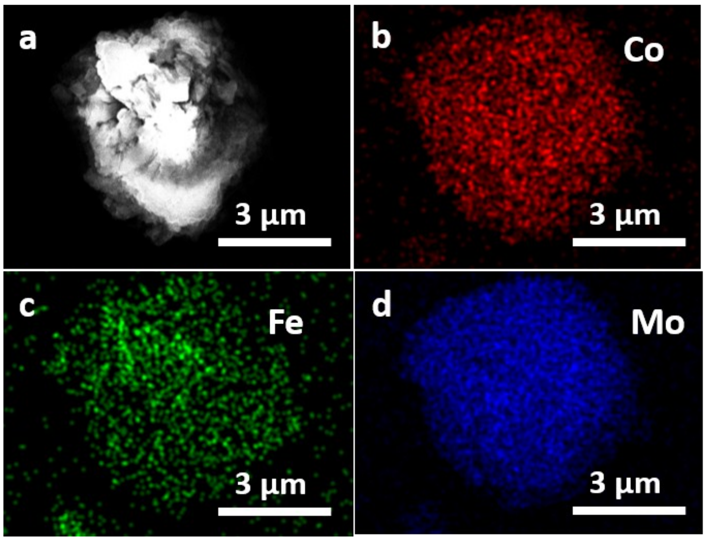

Fig. S3 EDS mapping images of initial FeCoMo sample.

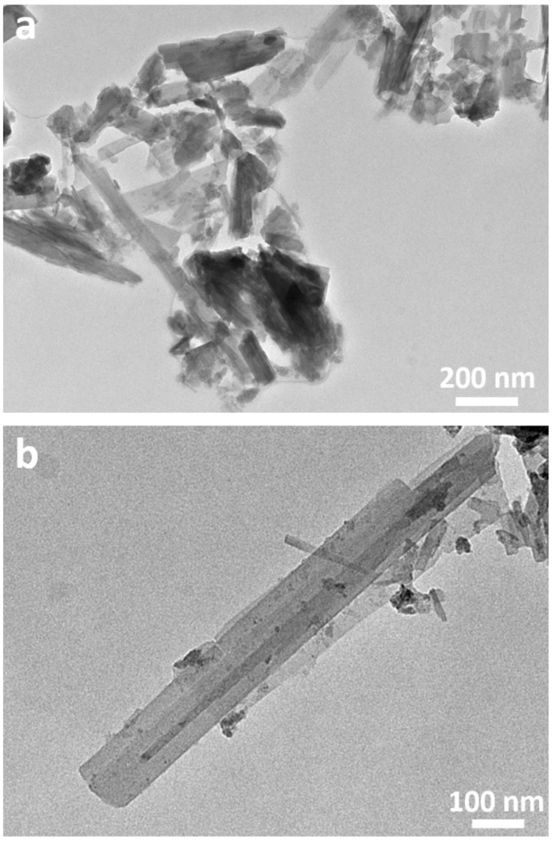

Fig. S4 TEM images of FeCoMo sample at different magnifications.

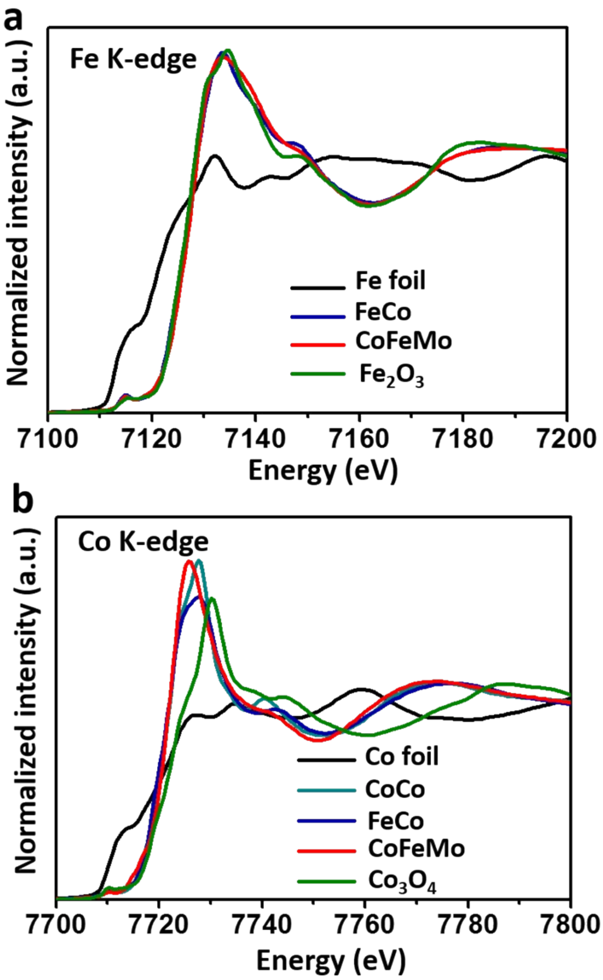

Fig. S5 The XAENS spectra of (a) Fe K-edge and (b) Co K-edge.

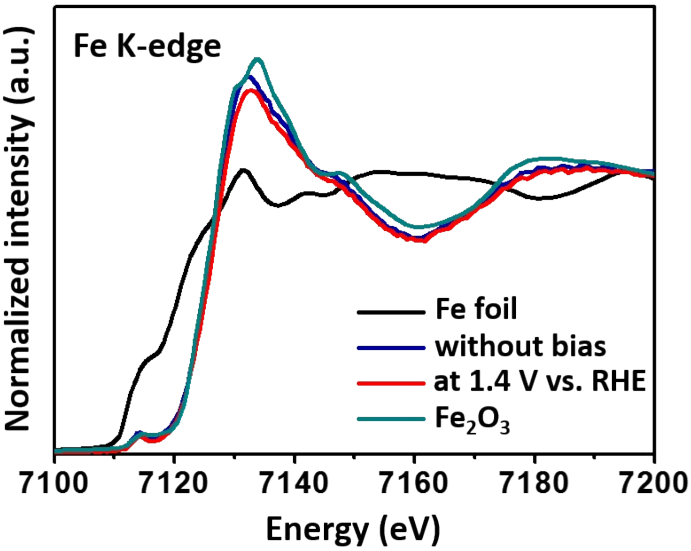

Fig. S6 The *in-situ* XAENS spectra of Fe K-edge for the FeCoMo sample with and without bias.

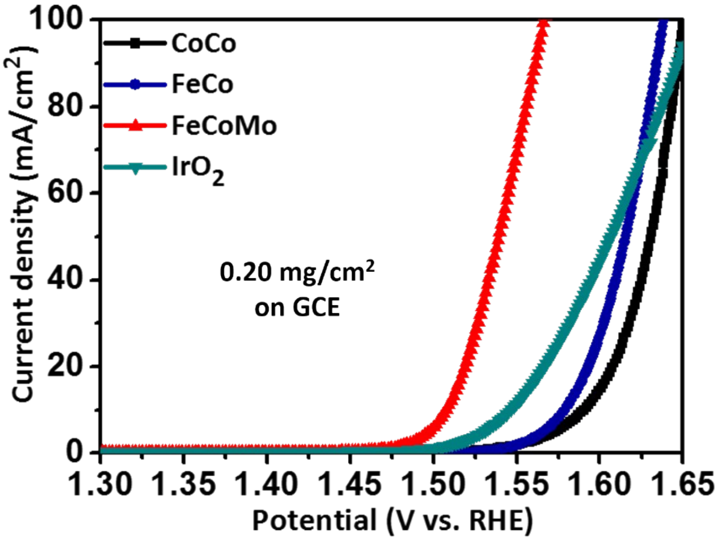

Fig. S7 The LSV curves of CoCo, FeCo, FeCoMo and IrO_2 samples, respectively.

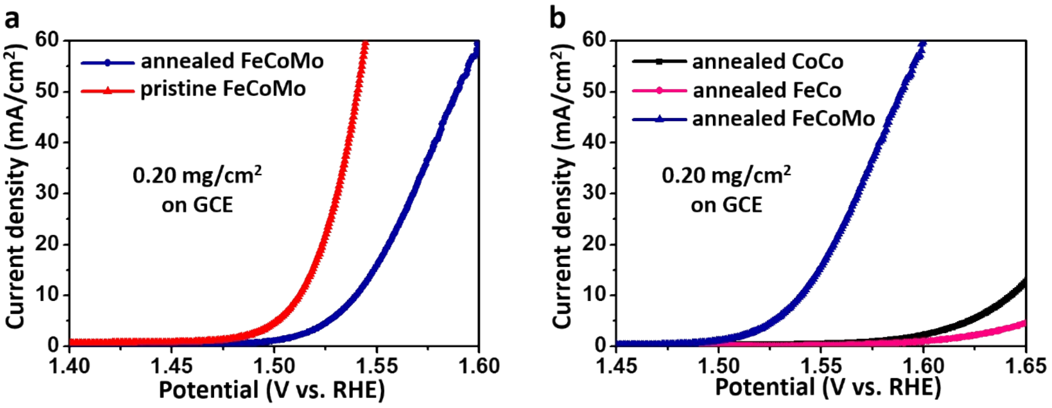

Fig. S8 LSV curves of (a) annealed and pristine FeCoMo samples, and (b) annealed CoCo, FeCo and FeCoMo samples.

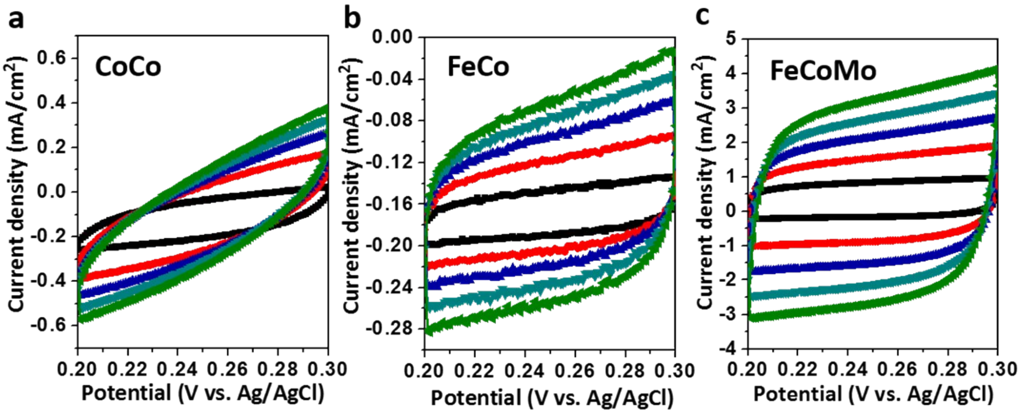

Fig. S9 Cyclic voltammograms of CoCo, FeCo and FeCoMo at different scan rates (from 20 to 180 mV/s with an increment of 40 mV/s).

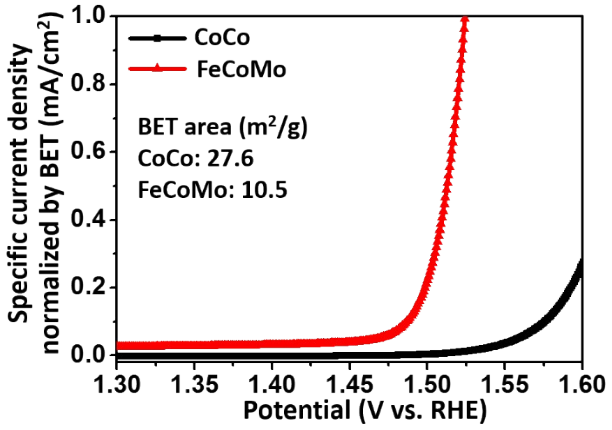

Fig. S10 The OER polarization curve of FeCoMo catalyst and controlled CoCo sample in three-electrode configuration in 1 M KOH aqueous electrolyte loaded on GCE normalized by BET surface areas.

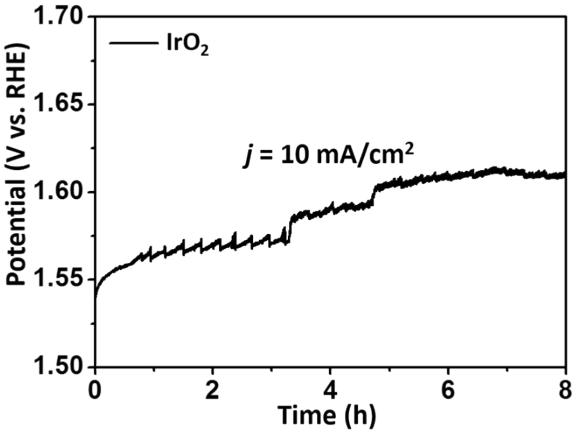

Fig. S11 The stability test of IrO_2 in 1 M KOH.

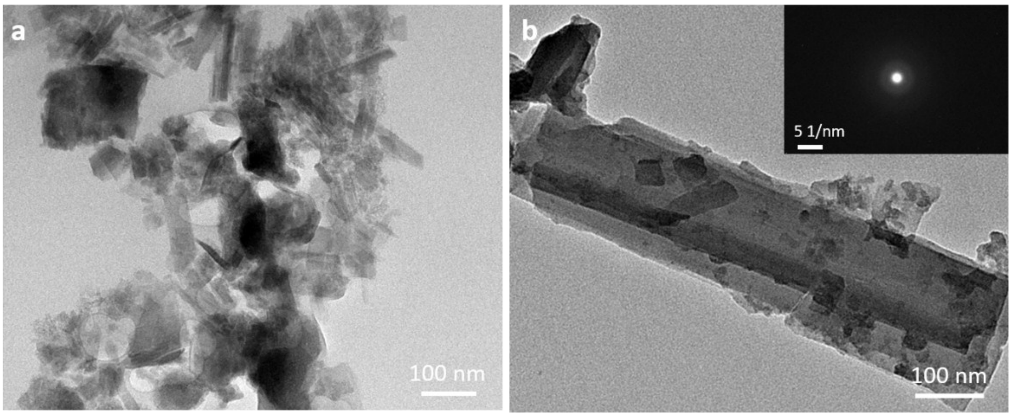

Fig. S12 The TEM images of FeCoMo sample after OER stability test for 40 h. Inset of (b): the SAED pattern of FeCoMo sample.

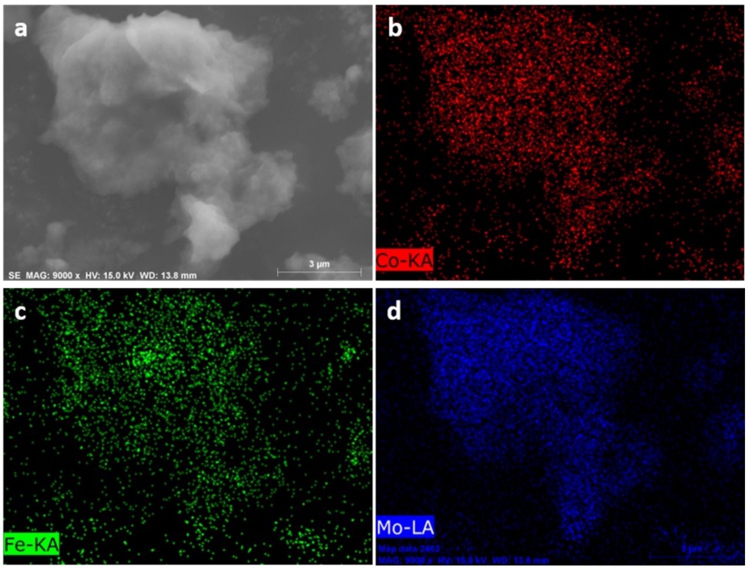

Fig. S13 Element mapping images of FeCoMo samples after OER test for 30 h.

Table S1 Comparison of OER catalytic parameters of recently reported Co and FeCo based OER materials.

**Catalysts**[a]	**η_{10} (mV)**[b]	**j_{300} (mA/cm^2)**[c]	**Tafel slope (mV/dec)**	**Bulk mass activity (A/g)**[d]	**References**
FeCoMo	277	35.5	27.74	177.35	this work
CoCo	361	0.9	56.06	4.4	this work
FeCo	349	0.5	41.51	2.3	this work
IrO_2	316	5	48.78	25.2	this work
CoCo NS	353	0.744	59	10.6	Ref. 1
N-doped graphene-CoO	340	~3	71	4.3	Ref. 2
CoPi	380	~1	58.7	5.0	Ref. 3
Co-Bi NS/G	290	~15	53	52.6	Ref. 4
Co-UMOFNs	371	0.1	103	0.5	Ref. 5
α-$Co_4Fe(OH)_x$	295	~12	52	42.9	Ref. 6
$CoFe_2O_4$/PANI-MWCNTs	314	~5	30.69	17.5	Ref. 7
CoFe/C	300	~8	61	/	Ref. 8
Co-Fe-O/rGO	340	~1	31	10.0	Ref. 9

[a] All the catalysts were deposited on the GCE to evaluate the OER performances; [b] η_{10} means the overpotential to achieve the current density of 10 mA/cm^2; [c] j_{300} means the current density at the overpotential of 300 mV; [d] The bulk mass activity was calculated at the overpotential of 300 mV based on the loading amount.

Table S2 The ICP information of leached Mo^{6+} in the electrolyte.

Conditions	Time (h)	Mo^{6+} concentration (mg/L)	Leached Mo^{6+} (mg)
biased at 1.53 V	70	< 0.040	< 0.006
without bias	240	< 0.040	< 0.006

Notes: The OER test was performed with a glassy carbon electrode (GCE, with 5 mm in diameter and 0.197 cm^2 in area) as the working electrode, the Ag/AgCl (3.5 M KCl solution) electrode as the reference electrode and a graphite rod (spectral purity, 3 mm in diameter) as the counter electrode, with 1 M KOH (pH 13.6) as the electrolyte. The total loading amount of the catalyst is about 0.0394 mg and the total volume of the electrolyte is about 150 mL. Then, the FeCoMo electrode was biased at 1.50 V (vs. RHE) to generate oxygen for more than 70 hours and also stayed in the electrolyte without bias for more than 10 days. After the treatment, the electrolyte was extracted and dissolved with concentrated H_2SO_4 for analysis. Negligible leaching phenomenon was found both in the working condition and the condition without bias in 1 M KOH.

References

1. F. Song and X. Hu, *Nat. Commun.*, 2014, **5**, 4477.
2. S. Mao, Z. Wen, T. Huang, Y. Hou and J. Chen, *Energy Environ. Sci.*, 2014, **7**, 609-616.
3. M. Pramanik, C. Li, M. Imura, V. Malgras, Y.-M. Kang and Y. Yamauchi, *Small*, 2016, **12**, 1709-1715.
4. P. Chen, K. Xu, T. Zhou, Y. Tong, J. Wu, H. Cheng, X. Lu, H. Ding, C. Wu and Y. Xie, *Angew. Chem. Int. Ed.*, 2016, **55**, 2488-2492.
5. S. Zhao, Y. Wang, J. Dong, C.-T. He, H. Yin, P. An, K. Zhao, X. Zhang, C. Gao, L. Zhang, J. Lv, J. Wang, J. Zhang, A. M. Khattak, N. A. Khan, Z. Wei, J. Zhang, S. Liu, H. Zhao and Z. Tang, *Nat. Energy*, 2016, **1**, 16184.
6. H. Jin, S. Mao, G. Zhan, F. Xu, X. Bao and Y. Wang, *J. Mater. Chem. A*, 2017, DOI: 10.1039/C6TA09959A.
7. Y. Liu, J. Li, F. Li, W. Li, H. Yang, X. Zhang, Y. Liu and J. Ma, *J. Mater. Chem. A*, 2016, **4**, 4472-4478.
8. B. Ni and X. Wang, *Chem. Sci.*, 2015, **6**, 3572-3576.
9. J. Geng, L. Kuai, E. Kan, Q. Wang and B. Geng, *ChemSusChem*, 2015, **8**, 659-664.
